# Behavioral intention of mental health practitioners toward the adoption of virtual humans in affect recognition training

**DOI:** 10.3389/fpsyg.2022.934880

**Published:** 2022-10-12

**Authors:** Arturo S. García, Patricia Fernández-Sotos, Pascual González, Elena Navarro, Roberto Rodriguez-Jimenez, Antonio Fernández-Caballero

**Affiliations:** ^1^Unidad Multidisciplinar de Investigación de la Neurocognición y Emoción en Entornos Virtuales y Reales, Instituto de Investigación en Informática de Albacete, Albacete, Spain; ^2^Departamento de Sistemas Informáticos, Universidad de Castilla-La Mancha, Albacete, Spain; ^3^Servicio de Salud Mental, Complejo Hospitalario Universitario de Albacete, Albacete, Spain; ^4^Biomedical Research Networking Center in Mental Health (CIBERSAM), Madrid, Spain; ^5^Cognición y Psicosis, Area de Neurociencias y Salud Mental, Instituto de Investigación Sanitaria Hospital 12 de Octubre (imas12), Madrid, Spain; ^6^CogPsy-Group, Universidad Complutense de Madrid, Madrid, Spain

**Keywords:** affect recognition, mental health, social cognition, technology acceptance, UTAUT2, virtual humans

## Abstract

This paper explores the key factors influencing mental health professionals' behavioral intention to adopt virtual humans as a means of affect recognition training. Therapies targeting social cognition deficits are in high demand given that these deficits are related to a loss of functioning and quality of life in several neuropsychiatric conditions such as schizophrenia, autism spectrum disorders, affective disorders, and acquired brain injury. Therefore, developing new therapies would greatly improve the quality of life of this large cohort of patients. A questionnaire based on the second revision of the Unified Theory of Acceptance and Use of Technology (UTAUT2) questionnaire was used for this study. One hundred and twenty-four mental health professionals responded to the questionnaire after viewing a video presentation of the system. The results confirmed that mental health professionals showed a positive intention to use virtual reality tools to train affect recognition, as they allow manipulation of social interaction with patients. Further studies should be conducted with therapists from other countries to reach more conclusions.

## 1. Introduction

Social cognition addresses how people process, store, and apply information about other people and social situations (Brothers, 1990; Pinkham et al., 2014). Social cognition can be described as “the ability to construct representations of relationships between oneself and others, and to use those representations flexibly to guide social behaviors” (Adolphs, 2003; Brown et al., 2012). Impaired social cognition is a core feature of several psychiatric illnesses, the paradigmatic illness being schizophrenia. People who suffer from schizophrenia present severe deficits in their daily functionality, due in part to this social cognition deterioration (Couture et al., 2006). According to several studies, social cognition could have a greater influence than neurocognition on functionality, or even act as a mediating variable between neurocognition and functionality (Fett et al., 2011; Schmidt et al., 2011). Functional impairments are manifested in various areas such as maintaining interpersonal relationships, the ability to be independent for activities of daily living, and the performance of work, pleasure, and leisure activities (Bellack et al., 2006; Green et al., 2008; Lahera et al., 2012). Thus, achieving an adequate level of functioning has become one of the main objectives of psychiatric rehabilitation.

Social cognition is divided into four relatively independent domains: theory of mind, attributional style, social perception, and emotional processing (Pinkham et al., 2016; Fernández-Sotos et al., 2018, 2019). Emotional processing refers to the ability to perceive, recognize and manage emotional information (Green et al., 2008). It is also defined as the ability to identify, facilitate, regulate, understand, and manage emotions (Mayer et al., 2001). This domain is further divided into three subdomains (Pinkham et al., 2014) that include lower and higher level processes. Higher level processes encompass understanding and emotional management while the lower perceptual level includes facial affect recognition. Facial affect recognition is described as the identification and recognition of emotional states through facial expressions and/or non-facial cues such as voice (Pinkham et al., 2014). This capacity is used daily by individuals and is crucial for effective social interaction, determining a large part of social functioning (Johnston et al., 2008). Therefore, the way in which an individual recognizes the emotional state in another affects his/her social success, which is relevant for adaptation in the community (Sachs et al., 2012).

There is consistent evidence that patients with schizophrenia have significant difficulty accurately recognizing emotions expressed by others (Marwick and Hall, 2008). This deficit can generate a misinterpretation of social situations and, therefore, a significant deficit in social functioning (Bordon et al., 2017). Due to the relevance for social functioning in these patients and for quality of life, different interventions have been designed to improve facial affect recognition. Recent meta-analyses have shown promising results of these psychotherapeutic approaches in terms of facial affect recognition and functionality (Kurtz and Richardson, 2011; Bordon et al., 2017).

Traditionally, therapies aimed at remediating facial affect recognition deficits used pictures showing a range of emotions. However, facial movements, voice, and gestures are important cues for the recognition (Roark et al., 2003; Garrido et al., 2016; Pan et al., 2021; Tiwari et al., 2021) and regulation (Colombo et al., 2019; Shan et al., 2021; Wang et al., 2022) of affect. Therefore, virtual humans (VHs) overcome many restrictions on the use of static images through animation, or even videos, by offering an unlimited set of designs (avatars in the social context) to practitioners (Fernández-Caballero et al., 2017; García et al., 2018; del Aguila et al., 2021; Zhang et al., 2021). However, although virtual reality technology has been applied to treat communication disabilities (Bailey et al., 2021), autism spectrum disorder (Cai et al., 2013; Kuriakose and Lahiri, 2015), and psychotic disorders (Taubneblatt et al., 2019), among others, it still remains novel to many therapists.

The objective of this paper was to study the willingness of therapists to use this type of tool. Therefore, this article studied the factors that influence the behavioral intention of mental health practitioners to adopt the proposed technology through the screening of a video presenting a software tool. This stool, managed by the therapists, was designed for the remediation of affect recognition deficits (Garćıa et al., 2019). To study such willingness, the clinicians had to complete a questionnaire based on the Extended Unified Theory of Acceptance and Use of Technology (UTAUT2) (Venkatesh et al., 2012). UTAUT2 is a framework that outlines seven constructs—performance expectancy (PE), effort expectancy (EE), social influence (SI), facilitating conditions (FC), hedonic motivation (HM), price value (PV), and habit (H)—that play a decisive role in behavioral intention (BI) and use behavior (UB) in adopting a new technology.

The main contribution of this research was to engage a group of mental health professionals to study the factors that influence their decision to use remotely controlled VHs in the treatment of social cognition deficits. This technology is currently limited to research and is not available to the majority of therapists, so knowing their opinions could help to improve the tools and make them more accessible to therapies with patients.

The hypotheses analyzed in this work were:

- H1: Performance expectancy of the VH tool for the remediation of affect recognition deficits will positively influence therapists' behavioral intention to use the technology.
- H2: Effort expectancy of the use of the VH tool will not affect the therapists' behavioral intention to use it.
- H3: Social influence will not affect the therapists' behavioral intention to use the VH tool for their affect recognition therapies.
- H4: Facilitating conditions will positively affect the therapists' behavioral intention to use the technology.
- H5: Hedonic motivation will positively affect the therapist's behavioral intention to use VH tool.

## 2. Materials and methods

The present study was based on the UTAUT2 model as an alternative and/or complement to another relevant acceptance model such as the Technology Acceptance Model (TAM) (Tseng et al., 2013; Bassfar, 2021; Yan et al., 2022). UTAUT and UTAUT2 is also being extensively used in recent years in health care (Lin et al., 2016; Sezgin et al., 2018; Juan-González et al., 2021). PV, H, and UB were not included in our model because their measurement is not clear in this domain. Table 1 shows the questions posed for all the constructs analyzed.

**Table 1:** UTAUT2 survey items.

	**Performance Expectancy**
PE1.	The use of the VH tool presented in the video would be useful in my
	daily work with patients.
PE2.	Using this VH tool would increase the chances of making progress in
	patient's affect recognition.
PE3.	Using this VH tool would help me to achieve the objectives of the
	therapies quicker.
PE4.	Using this VH tool would increase my productivity.
	**Effort Expectancy**
EE1.	Learning how to use this VH tool is easy.
EE2.	The interaction with this VH tool is clear and understandable.
EE3.	I find this VH tool easy to use.
EE4.	It would be easy for me to become skillful at using this VH tool.
	**Social Influence**
SI1.	People who are important to me think that I should use this kind of
	VH tool.
SI2.	People who influence my behavior think that I should use this kind
	of VH tool.
SI3.	People whose opinions I value prefer that I use this kind of VH tool.
	**Facilitating Conditions**
FC1.	I have the resources necessary to use this kind of VH tool.
FC2.	I have the knowledge necessary to use this kind of VH tool.
FC3.	This VH tool is compatible with other systems or ways of designing
	therapies that I know.
FC4.	I could get help from others if I have difficulties using this kind of
	VH tool.
	**Hedonic Motivation**
HM1.	The VH tool presented in the video looks funny.
HM2.	The VH tool presented in the video looks enjoyable.
HM3.	The VH tool presented in the video looks very entertaining.
	**Behavioral Intention**
BI1.	I would intend to use the VH tool presented in the video with my
	patients.
BI2.	I would use this VH tool in my daily work with patients.
BI3.	I would use this VH tool frequently.

A total of 124 mental health professionals participated in the study, including psychiatrists, clinical psychologists and clinical nurses specialized in mental health. All belonged to the Spanish Biomedical Research Networking Center in Mental Health. Table 2 summarizes the demographic data of the subjects. The sample was predominantly composed of females, which roughly resembles the ratio of female practitioners in mental health care in Spain. According to the Spanish Institute of Statistics, the share of women licensed in medicine was 53% in 2021 https://www.ine.es/jaxi/Tabla.htm?tpx=48995&L=0. In the case of psychology and nursing the figures rose to 81 and 84%, respectively.

**Table 2:** Demographic and descriptive statistics.

	**Freq**.	**%**
**Gender**		
Male	27	21.77
Female	97	78.23
**Age**		
23–35	66	53.23
36–61	58	46.77
**Experience in social cognition**		
Yes	40	32.26
No	84	67.74
**Profession**		
Psychiatrist	61	49.19
Clinical Psychologist	17	13.71
Mental Health Nurse	46	37.10

Participants were required to log in to YouTube to view the video and then to a web questionnaire developed using Google Forms. The first section provided a short introduction to the intended purpose of the study and a consent to participate. The second gathered participant demographic information, while the third collected use intention of the technology, as measured on a 7-point Likert scale (1 = strongly disagree, 2 = quite strongly disagree, 3 = slightly disagree, 4 = neither agree nor disagree, 5 = slightly agree, 6 = quite strongly agree, and 7 = strongly agree).

**Figure 1:**
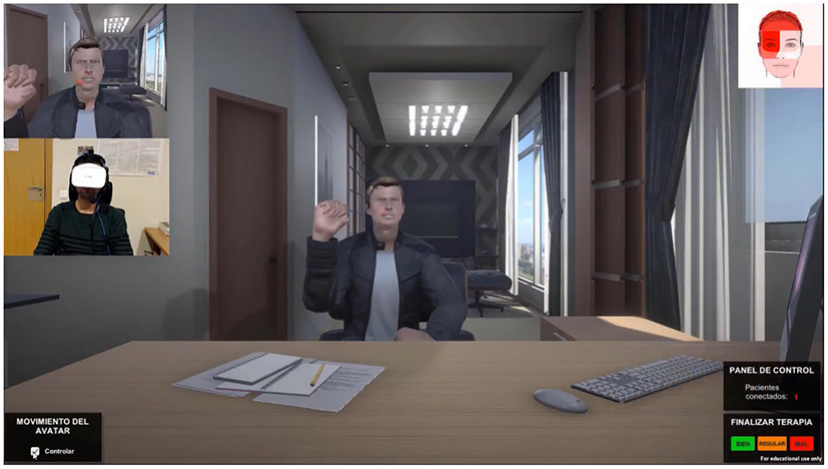
User interface of the tool shown to the therapists participating in the experiment.

The video presented to the participants was a recording of an acted performance of a therapy session using the multimodal avatar-based tool (Garćıa et al., 2019). Figure 1 depicts the user interface of the therapist's tool showing the avatar (center), the patient recorded by a webcam and the view from his/her perspective (top-left), as well as a visual representation of the time looking to each part of the avatar's face (top-right). The patient is immersed using a head mounted display, having the avatar in front of him/her. The therapist takes control of the avatar at any time, using his/her own body and facial movements to animate it, while the system modifies his/her voice to match the avatar's gender.

The hypotheses will be expanded in the following section when considering the moderation effects of age and experience.

## 3. Results

The results for both constructs and items were rated very positively by practitioners, as shown in Table 3. SmartPLS v.3.2.8 was used to implement the partial least squares structural equation modeling (PLS-SEM) algorithm with data collected from the questionnaire. Guidelines provided by other authors were adhered to for interpreting the results and evaluating the model (Hair et al., 2011, 2013, 2014, 2019; Henseler et al., 2016; Peng and Yan, 2022; Yan et al., 2022). These covered the evaluation of the measurement model, the structural model and moderation effects.

**Table 3:** Descriptive statistics for the constructs and items.

	**Mean**	**SD**	**Median**	**IQR**
**Performance Expectancy**	5.31	1.48	6	1
PE1	5.24	1.55	6	1
PE2	5.51	1.20	6	1
PE3	5.32	1.44	6	1
PE4	5.16	1.65	6	2
**Effort Expectancy**	5.67	1.10	6	1
EE1	5.69	1.13	6	1
EE2	5.61	1.05	6	1
EE3	5.65	1.13	6	1
EE4	5.75	1.12	6	1
**Social Influence**	4.12	1.99	4	4
SI1	4.12	1.99	4	4
SI2	4.03	1.89	4	4
SI3	4.12	1.95	4	3.25
**Facilitating Conditions**	5.07	1.73	6	2
FC1	4.56	2.15	6	3
FC2	4.99	1.70	6	2
FC3	5.40	1.37	6	1
FC4	5.35	1.49	6	1
**Hedonic Motivation**	5.50	1.52	6	1
HM1	5.50	1.46	6	1
HM2	5.51	1.37	6	1
HM3	5.48	1.50	6	1
**Behavioral Intention**	5.51	1.66	6	2
BI1	5.50	1.74	6	2
BI2	5.26	1.84	6	3
BI3	5.23	1.82	6	1

### 3.1. Measurement model

Prior to testing the hypotheses, the measurement items of the questionnaire were analyzed for construct reliability and validity. Since it is a reflective model—the indicators represent a consequence of the construct, instead of a cause (Rossiter, 2002)—, factor reliability, internal consistency, convergent validity and discriminant validity were tested. In general, the higher the factor loadings, the stronger and more reliable the measurement model. The factor loadings are shown in the diagonal of Table 4. All standardized factor loadings were above the threshold of 0.70, which suggests that the scales are reliable. The square of the factor loadings was also greater than 0.5 for all items.

**Table 4:** Factors loadings and cross-loadings.

	**BI**	**EE**	**FC**	**HM**	**PE**	**SI**
**BI1**	0.97	0.64	0.69	0.86	0.87	0.57
**BI2**	0.97	0.61	0.71	0.87	0.88	0.55
**BI3**	0.98	0.61	0.71	0.84	0.88	0.58
**EE1**	0.47	0.88	0.47	0.43	0.38	0.41
**EE2**	0.58	0.92	0.53	0.56	0.52	0.42
**EE3**	0.57	0.93	0.54	0.53	0.46	0.37
**EE4**	0.66	0.89	0.59	0.63	0.53	0.50
**FC1**	0.68	0.46	0.90	0.65	0.67	0.58
**FC2**	0.67	0.64	0.90	0.65	0.64	0.52
**FC3**	0.54	0.44	0.76	0.55	0.51	0.38
**FC4**	0.57	0.48	0.84	0.55	0.51	0.46
**HM1**	0.82	0.54	0.66	0.92	0.74	0.53
**HM2**	0.83	0.56	0.66	0.95	0.77	0.52
**HM3**	0.85	0.60	0.67	0.95	0.79	0.48
**PE1**	0.87	0.47	0.64	0.76	0.93	0.56
**PE2**	0.72	0.43	0.51	0.69	0.87	0.46
**PE3**	0.82	0.49	0.65	0.74	0.93	0.56
**PE4**	0.88	0.53	0.70	0.77	0.92	0.54
**SI1**	0.56	0.46	0.55	0.52	0.57	0.98
**SI2**	0.54	0.45	0.52	0.49	0.54	0.97
**SI3**	0.60	0.47	0.61	0.57	0.59	0.97

Grey color indicates the values of the table's diagonal.

The *internal consistency* of the constructs were checked using composite reliability and Cronbach's alpha (see Table 5). Both coefficients exceeded the threshold of 0.70. As the next step, *convergent validity* was corroborated using AVE (average variance extracted), being above 0.50 (Table 5). Therefore, at least 50% of the variance is explained by the construct items, which offers adequate evidence of reliability.

**Table 5:** Internal consistency, construct reliability, and convergent validity.

	**Cronbach's**	**Composite**	**Average variance**
	**alpha**	**reliability**	**extracted (AVE)**
BI. Behavioral Intention	0.97	0.98	0.94
EE. Effort Expectancy	0.93	0.95	0.82
FC. Facilitating Conditions	0.87	0.91	0.73
HM. Hedonic Motivations	0.94	0.96	0.89
PE. Performance Expectancy	0.93	0.95	0.83
SI. Social Influence	0.97	0.98	0.94
Thresholds	≥0.70	≥0.70	≥0.50

Finally, *discriminant validity* deals with the degree to which a construct is empirically distinguishable from other constructs in the structural model. The Fornell-Larcker criterion has conventionally been used to assess discriminant validity. This criterion compares the square root of the AVE values with the correlations of the latent variables, which must be greater than their highest correlation with any other construct (see Table 6).

**Table 6:** Fornell-Larcker criterion table.

	**BI**	**EE**	**FC**	**HM**	**PE**	**SI**
BI	0.971					
EE	0.639	0.905				
FC	0.726	0.593	0.853			
HM	0.882	0.603	0.707	0.941		
PE	0.901	0.528	0.688	0.815	0.913	
SI	0.583	0.473	0.576	0.543	0.584	0.971

The square root of AVE is shown in the diagonal cells and the correlation are presented just below.

However, the use of this criterion has declined, as the heterotrait-monotrait (HTMT) ratio has been shown to work better (Henseler et al., 2015). Therefore, the latter approach was applied to confirm discriminant validity, which is accepted if this statistic is different from 1. HTMT_0.90_ confirmed that there were no discriminant validity issues between any pair of constructs other than BI and HM, and BI and PE (see Table 7). In this case, the HTMT ratios were slightly above the threshold established by HTMT_0.90_ (0.926 and 0.942, respectively). Nevertheless, the more conservative HTMT_inference_ approach has been suggested for technology acceptance models (Henseler et al., 2015). Attending to this criterion, the confidence interval (CI) did not include value 1 and thus confirmed the discriminant validity of these pairs of constructs (CI_0.95_ [0.877;0.961] for BI and HM, and CI_0.95_ [0.904;0.969] for BI and PE).

**Table 7:** Heterotrait-monotrait ratio (HTMT) values.

	**BI**	**EE**	**FC**	**HM**	**PE**	**SI**
BI						
EE	0.664					
FC	0.785	0.651				
HM	0.926	0.637	0.780			
PE	0.942	0.558	0.752	0.871		
SI	0.599	0.494	0.618	0.569	0.610	

### 3.2. Structural model

As the measurement model was found valid and reliable, the next step focused on the evaluation of the structural model. This included calculating the coefficient of determination (R^2^), path coefficients, effect size (f^2^) and the predictive relevance of the model (Q^2^), which are standard assessment criteria for evaluating the inner structural model. Collinearity increases standard errors, makes unreliable significance testing of independent variables, and prevents the researcher from assessing the relative importance of one independent variable compared to another (Hair et al., 2016). Variance inflation factors (VIF) were calculated to test for collinearity. All of them were less than 4, the cut-off value (Garson, 2016) (see Table 8). This ruled out collinearity as a critical issue in the model, allowing the evaluation and interpretation of structural relationships.

**Table 8:** Collinearity.

	**BI**
BI	
EE	1.75
FC	2.47
HM	3.62
PE	3.39
SI	1.70

Assessment of variance inflation factors (VIF) of the constructs.

**Figure 2:**
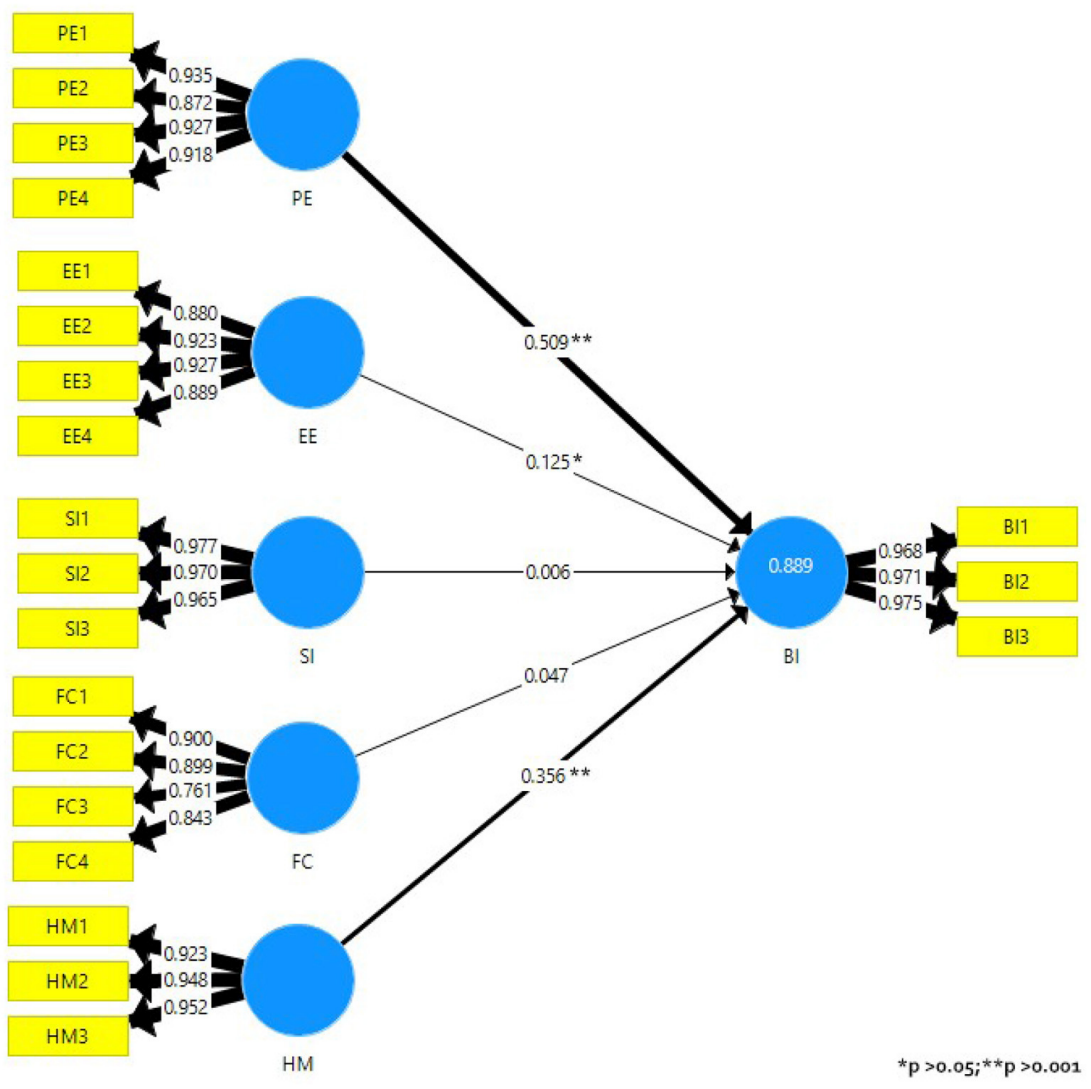
Results of the structural model.

The path coefficients representing the relationships among constructs are plotted in Figure 2. The higher the path coefficient, the greater the substantial effect of the exogenous construct on the endogenous latent construct. EE → BI (0.125, *p* < 0.05), HM → BI (0.356, *p*>0.001) and PE → BI (0.509, *p*>0.001) were found significant using the bootstrapping procedure (10, 000 sub-samples). Therefore, it could also be stated that the effects of EE on BI were lower than the ones of HM and PE, which are moderate and strong, respectively.

The predictive accuracy of the model is measured by the coefficient of determination R^2^. This indicates the amount of variance of a construct, explained by the predictor variables of the endogenous construct, whose values range from 0 to 1. There was only one endogenous variable in this model, so R^2^ for BI was 0.889. Even though the interpretation of R^2^ depends on the discipline under study, the rule of thumb is that its effects are described as substantial, moderate, or weak for values of 0.75, 0.50, and 0.25, respectively (Hair et al., 2011). Therefore, it was concluded that the model explains 88.9% of the variance in BI.

A cross-validated redundancy Q^2^ value larger than 0 means that the exogenous constructs have predictive relevance for the endogenous construct under consideration (Hair et al., 2011). In other words, the model is relevant to predicting that factor. The value of Q^2^ was 0.779. Values higher than 0, 0.25, and 0.5 depict small, medium and large predictive accuracy, respectively, of the PLS path model (Hair et al., 2019). As a conclusion, both R^2^ and Q^2^-values demonstrated that the model had substantial predictive precision and relevance in relation to the BI construct. Furthermore, the path coefficients indicate that the only significant effects of the constructs on BI were caused by PE, EE, and HM.

### 3.3. Moderation

**Figure 3:**
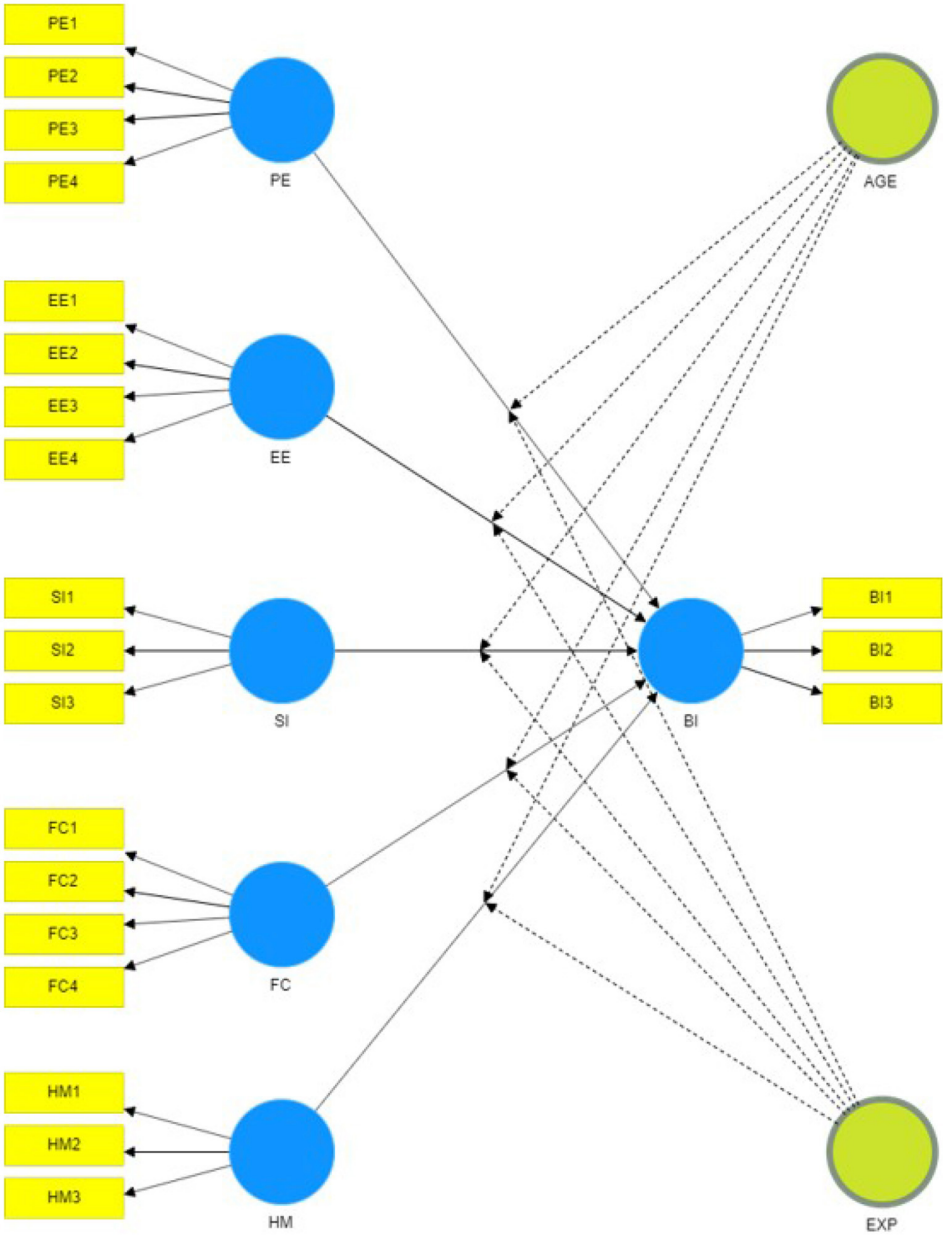
Structural model including age and experience as moderator variables. The image has been adapted to show which relationships are expected to be affected by which moderating variable.

We re-ran the tests including moderation to test the remaining hypotheses. Multigroup analysis was discarded due to the difference in group size and the low sample size of some of the groups (see demographic information on Table 2). Therefore, a two-stage moderation approach was adopted. In fact, we wanted to give priority to the detection of a significant interaction, and the sample size made us focus on statistical power (Hair et al., 2016). Moreover, dummy variables were added as in previous works (Henseler and Fassott, 2010; Hair et al., 2016). Figure 3 shows the inclusion of the moderator variables in the model.

In accordance with a recent brief research report on the acceptance of Information and Communications Technology (ICT) (Nunes et al., 2019), age (AGE) was expected to moderate the effect that all exogenous constructs have on BI. Nunes et al. (2019) recalled that age seems to have a key moderating effect on the BI, and the effects of PE are stronger for younger rather than older adults. For older adults it is EE, SI and FC that would stand out. Age was divided into two groups bounded by the 35-year median age of the participants. Similarly, experience (EXP) was expected to moderate the effect of PE, SI, and FC on BI. Here, it was supposed that therapists with experience in classical social cognition therapies would be more interested in using the new tools, as they would be able to see the added value of applying the technology to their field of expertise. All this led to eight new hypotheses (see Table 9).

**Table 9:** Moderation hypotheses.

**#**	**Influence**	**Type of effect expected**
H6 (a)	AGE^*^PE → BI	Stronger for the younger group
H6 (b)	AGE^*^EE → BI	Stronger for the younger group
H6 (c)	AGE^*^SI → BI	No moderation effect expected
H6 (d)	AGE^*^FC → BI	Stronger for the younger group
H6 (e)	AGE^*^HM → BI	Stronger for the younger group
H7 (a)	EXP^*^PE → BI	Stronger for the experienced group
H7 (b)	EXP^*^SI → BI	Stronger for the not experienced group
H7 (c)	EXP^*^FC → BI	Stronger for the experienced group

#### 3.3.1. H6 age moderation

The moderation effects of the categorical dummy variable accounting for the two age groups were studied, since differences between the younger and older groups were expected (interaction effects of age on the relationships of the constructs). The moderating pathways of the FC → BI and HM → BI relationships were found to be significant (−0.133, *p* < 0.05 and 0.141, *p* < 0.05). This suggested that the relationship of FC → BI becomes stronger with low levels of age, supporting H6(d). However, it was not the case for HM → BI, as it became stronger with high levels of age, not supporting H6(e). H6(c) was confirmed, as age was not expected to moderate the relationship of SI with BI. The remaining H6 hypotheses groups were not supported because the paths were not significant.

#### 3.3.2. H7 experience moderation

Similarly, participants' experience was coded into a dummy variable (yes/no) to perform the moderation analysis. Again, the results showed no interaction effects of experience on the relationship of the constructs with BI, rejecting hypothesis group H7.

## 4. Discussion and conclusion

The principal aim of this work was to explore the impact of certain key factors on the adoption of a therapist-controlled software tool for remediation of affect recognition deficits. A video demonstrating the characteristics of this tool was shown to 124 therapists. Upon viewing the video, they were instructed to complete a UTAUT2 model-based questionnaire. The collected data were analyzed using PLS-SEM to evaluate the model and test nine hypotheses.

Regarding the influence of the exogenous constructs on the BI endogenous construct, the results supported a positive influence of PE on BI. Over recent years, different rehabilitation therapies aimed at improving facial affect recognition have been developed, mainly in the area of schizophrenia. Many of them used virtual reality (VR) as core training tool, because it provides environments and situations practically similar to reality, by using dynamic avatars enabling social interaction with the participant, and manipulated to represent different emotional states (Gutiérrez-Maldonado et al., 2014). Several recent meta-analyses have reported promising results of psychotherapeutic approaches regarding facial emotion recognition and functionality (Kurtz and Richardson, 2011; Bordon et al., 2017). More specifically, a recent review in the field of VR and psychosis suggested that VR-based interventions are not only effective, but also well-tolerated by patients (Rus-Calafell et al., 2018). However, contrary to what was expected, EE had a low positive influence on BI. The effect was not as strong compared to PE. Actually, society has been using new technologies in a standardized way for the last 10 years, which reduces the fears of their complexity and the rejection of their use. In addition, the proposed tool should be effortless to use. Moreover, SI showed not to affect BI. Currently, mental health practitioners do not perceive that the most influential social environment expects that they will use this technology in clinical practice. The lack of standardized therapies aimed at improving social cognition probably influenced the results obtained. Likewise, FC did not influence BI. This was expected mainly because of the general lack of availability of VR equipment in Spanish hospitals. However, positive responses to questions FC2 and FC3 on the knowledge needed to use VR tools and their compatibility with other ways of designing therapies may have counteracted. Finally, as expected, therapists found the use of this technology enjoyable and entertaining, which is positive as it can be pleasant for patients as well, making therapy something they do not consider hard work.

In relation to the moderating effects that age and experience might have on the relationship of exogenous to endogenous constructs, several results were found. Younger participants thought they had the skills to use a tool like that described in the video, while older participants believed they would enjoy using such a tool more. As expected, age did not influence the relationship of SI and BI. As for the moderating effect that therapist experience in social cognition remediation had on the relationships in our model, no interaction effects were found. In recent years, social cognition in schizophrenia has taken a relevant role in the mental health area. This is probably related to its close relationship with functionality and quality of life (Fett et al., 2011; Schmidt et al., 2011). VR has also recently become an important alternative in the field of psychosis, with promising results (Rus-Calafell et al., 2018). A feasible explanation for the results obtained might be related to evidence that a significant proportion of mental health clinicians consider these deficits relevant and acknowledge the development of therapies aimed at their treatment, including those based on VR.

Lastly, the findings of this study suggest clinical implications for the use of this new technology in affect recognition training, as may be inferred from the therapists' intention to use it. However, several potential limitations in the interpretation of the results need to be kept in mind and addressed in future work. The first limitation concerns the size of the sample. Only 124 therapists participated in the video-based assessment and, although this is a representative sample of professionals from various Spanish regions, the inclusion of a larger number of therapists would be essential to draw further conclusions. In addition, an extension of this study to other countries would be interesting for a broader view on the acceptance of VH-based tools in affect recognition training. Another limitation has been the use of a video to demonstrate the tool rather than providing a hands-on experience. Although previous research could not find significant differences between video-based and live assessments (Woods et al., 2006), it seems reasonable to undergo a face-to-face validation process.

In terms of statistical evaluation, PLS-SEM was used to evaluate the results of the UTAUT2 model, but three-way and four-way interactions were not explored. These analyses would provide information on the impact that two or three moderating variables combined may have on the relationship of the exogenous constructs on the endogenous one. However, these types of interactions are difficult to estimate and interpret (Hair et al., 2013).

Finally, this research focused on one of two parties potentially interested in using a tool as described in this paper. Therefore, it would be worthwhile to collect the judgments of the patients, as they are the prominent counterpart intended to benefit from the tool. In this way, a complete picture of the acceptance of the technology by all parties involved should be obtained in the near future.

To overcome some of these limitations, future work will consider involving therapists in a practical session in which they can try out the real system, experiencing it first hand. In addition, we will aim to get feedback from some patients, as this will enable the improvement of the tool and make it more bearable for them.

## Data availability statement

The raw data supporting the conclusions of this article will be made available by the authors, without undue reservation.

## Ethics statement

The studies involving human participants were reviewed and approved by the institutional Ethics Committee of Complejo Hospitalario Universitario de Albacete in Spain. The participants provided written informed consent to participate in the study.

## Author contributions

AG and AF-C contributed to the conception and design of the study. PF-S, PG, EN, and RR-J developed the methodological and research aspects of the study. AG used the software and performed the statistical analysis. AG, PG, and EN wrote the first draft of the manuscript. All authors contributed to manuscript revision, read, and approved the submitted version.

## Funding

This study received funding from Spanish Agencia Estatal de Investigación. The funder was not involved in the study design, collection, analysis, interpretation of data, the writing of this article or the decision to submit it for publication. Grants PID2020-115220RB-C21, PID2019-108915RB-I0, and EQC2019-006063-P funded by MCIN/AEI/10.13039/501100011033 and by ERDF A way to make Europe. This work was also partially funded by CIBERSAM of the Instituto de Salud Carlos III (ISCIII) and co-funded by the European Union.

## Conflict of interest

The authors declare that the research was conducted in the absence of any commercial or financial relationships that could be construed as a potential conflict of interest.

## Publisher's note

All claims expressed in this article are solely those of the authors and do not necessarily represent those of their affiliated organizations, or those of the publisher, the editors and the reviewers. Any product that may be evaluated in this article, or claim that may be made by its manufacturer, is not guaranteed or endorsed by the publisher.
